# Ohmyungsamycins promote antimicrobial responses through autophagy activation via AMP-activated protein kinase pathway

**DOI:** 10.1038/s41598-017-03477-3

**Published:** 2017-06-13

**Authors:** Tae Sung Kim, Yern-Hyerk Shin, Hye-Mi Lee, Jin Kyung Kim, Jin Ho Choe, Ji-Chan Jang, Soohyun Um, Hyo Sun Jin, Masaaki Komatsu, Guang-Ho Cha, Han-Jung Chae, Dong-Chan Oh, Eun-Kyeong Jo

**Affiliations:** 1 Department of Microbiology, Chungnam National University School of Medicine, Daejeon, 35015 South Korea; 2 Department of Medical Science, Chungnam National University School of Medicine, Daejeon, 35015 South Korea; 3 Natural Products Research Institute, College of Pharmacy, Seoul National University, Seoul, 08826 South Korea; 4 Molecular Mechanism of Antibiotics, Division of Life Science, Research Institute of Life Science, Gyeongsang National University, Jinju, 52828 South Korea; 5 Department of Biochemistry, Niigata University Graduate School of Medical and Dental Sciences, Niigata, 9518510 Japan; 6 Department of Infection Biology, Chungnam National University School of Medicine, Daejeon, 35015 South Korea; 7 Department of Pharmacology, Chonbuk National University Medical School, Jeonju, 54907 South Korea

## Abstract

The induction of host cell autophagy by various autophagy inducers contributes to the antimicrobial host defense against *Mycobacterium tuberculosis* (Mtb), a major pathogenic strain that causes human tuberculosis. In this study, we present a role for the newly identified cyclic peptides ohmyungsamycins (OMS) A and B in the antimicrobial responses against Mtb infections by activating autophagy in murine bone marrow-derived macrophages (BMDMs). OMS robustly activated autophagy, which was essentially required for the colocalization of LC3 autophagosomes with bacterial phagosomes and antimicrobial responses against Mtb in BMDMs. Using a *Drosophila melanogaster*–*Mycobacterium marinum* infection model, we showed that OMS-A-induced autophagy contributed to the increased survival of infected flies and the limitation of bacterial load. We further showed that OMS triggered AMP-activated protein kinase (AMPK) activation, which was required for OMS-mediated phagosome maturation and antimicrobial responses against Mtb. Moreover, treating BMDMs with OMS led to dose-dependent inhibition of macrophage inflammatory responses, which was also dependent on AMPK activation. Collectively, these data show that OMS is a promising candidate for new anti-mycobacterial therapeutics by activating antibacterial autophagy via AMPK-dependent signaling and suppressing excessive inflammation during Mtb infections.

## Introduction

*Mycobacterium tuberculosis* (Mtb) is an important intracellular bacterial pathogen and the causative agent of human tuberculosis, which remains a serious global burden worldwide^1^. Mtb is able to survive in the hostile environment of host cells by preventing phagolysosomal fusion^2^. Autophagy is a self-digesting process that degrades cytoplasmic aggregates and damaged organelles to maintain homeostasis during metabolic and infectious diseases^3^. Accumulating evidence has suggested that antibacterial autophagy (xenophagy) is a cell-autonomous host defense that leads to antimicrobial responses against Mtb infections^4–8^. To date, numerous agents or signaling pathways have been shown to activate antibacterial autophagy against Mtb^4, 5, 9, 10^.

Among the autophagy-stimulating signals, we focused on the activation of AMP-activated protein kinase (AMPK), a key energy-sensing kinase in the maintenance of metabolic homeostasis and intracellular quality control in response to various stresses^11^. Our recent studies revealed that AMPK activation plays an important role in the antimicrobial responses against Mtb by inducing autophagy-related genes (ATG) and enhancing phagosomal maturation^12^. In addition, AMPK activation is often associated with inducing anti-inflammatory responses in immune cells by directly ameliorating pro-inflammatory signaling and limiting the synthesis of certain lipid intermediates relevant to inflammation^13–15^.

We previously reported that ohmyungsamycins (OMS) A and B are novel cyclic peptides that were isolated from a marine bacterial strain belonging to the *Streptomyces genus* collected from Jeju Island in Korea^16^. We previously showed that OMS-A and -B are cyclic peptides that exhibit inhibitory effects against a diverse range of cancer cells and bacteria such as *Bacillus subtilis*, *Kocuria rhizophila*, and *Proteus hauseri* ^16^. In this study, we demonstrated that OMS-A and -B are robust activators of autophagy that lead to antimicrobial responses against Mtb. We used a *Drosophila melanogaster (D. melanogaster)–Mycobacterium marinum* infection model to show that OMS treatment elicited anti-mycobacterial effects through autophagy activation *in vivo*. We further investigated the mechanisms by which OMS activated antimicrobial responses and showed that AMPK-dependent signaling was involved in the OMS-mediated activation of autophagy in murine bone marrow-derived macrophages (BMDMs). In addition, OMS treatment inhibited macrophage inflammatory responses during Mtb infection by activating the AMPK pathway. Together, these data suggest that the OMS-induced activation of autophagy and suppression of excessive pathologic inflammation may contribute to the innate host defenses against mycobacterial infection.

## Results

### OMS-A and OMS-B stimulate the killing of mycobacteria *in vitro* and *in vivo*

**Figure 1:**
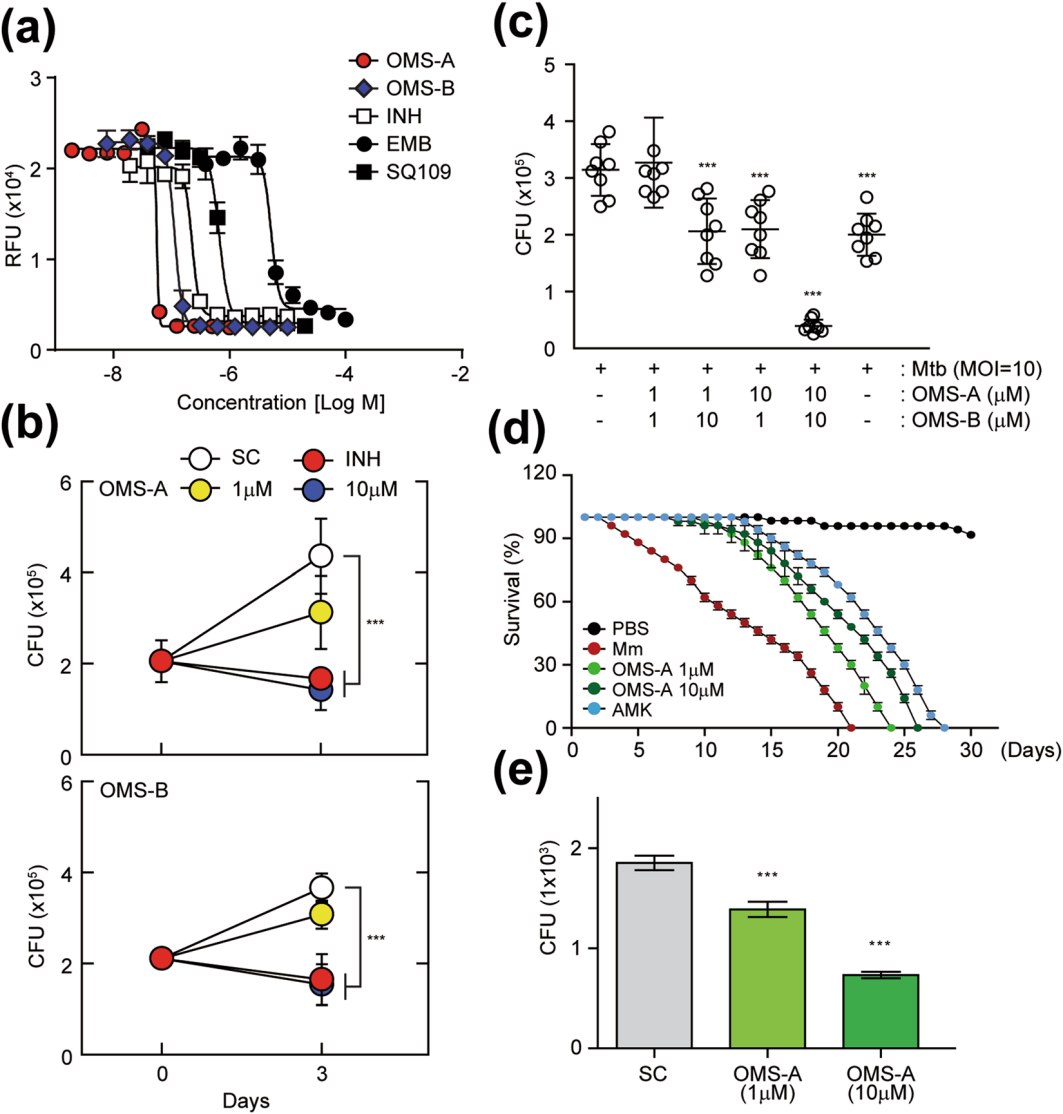
OMS shows antimycobacterial activity *in vitro* and *in vivo*. (**a**) Activity of OMS-A and OMS-B against Mtb replicating in culture broth medium. Mycobacterial growth was measured in relative fluorescence units (RFU). INH, EMB, and SQ-109 were used as positive controls. (**b**) BMDMs were infected with Mtb (moi = 10) for 4 h and then treated with OMS-A (1, 10 μM; *Top*), OMS-B (1, 10 μM; *Bottom*) or INH (0.5 μg/ml). (**c**) BMDMs were infected with Mtb and then co-treated with OMS-A and OMS-B or INH. (**b** and **c**) After 3 days, intracellular bacterial loads were determined by CFU assay. (**d**) *M. marinum* (CFU = 500)-infected W^1118^ flies were incubated with or without OMS-A (1, 10 μM) and AMK (1 μg/ml) medium. Dead flies were counted at 24 h intervals. The error bars indicate 95% confidence intervals. Log-rank analysis of the survival curves indicated that each group (n = 50) was significantly different. (**e**) W^1118^ flies were injected with *M. marinum* and then incubated with or without OMS-A (10 μM). After 12 days, each group (n = 20) of flies was harvested, homogenized, and quantified by CFU assay. All data represent the means ± SD of triplicates from each sample. ***p < 0.001, compared with Mtb-infected/untreated (**c**) and SC (**b**,**e**). INH, isoniazid; EMB, ethambutol; AMK, amikacin; SC, solvent control.

OMS-A and OMS-B have inhibitory activities against *Bacillus subtilis*, *Kocuria rhizophila*, and *Proteus hauseri* ^16^. To examine the anti-mycobacterial effects of OMS-A and OMS-B, we first assessed the antibacterial properties of OMS-A and OMS-B against Mtb. The minimum inhibitory concentration (MIC) of OMS-A and OMS-B against Mtb was determined using the resazurin microtiter assay (REMA) plate method. Isoniazid and ethambutol (known antimicrobial drugs) and SQ109 (an anti-Mtb drug candidate) were selected as positive controls^17^. After incubating Mtb for 5 days with the compounds, a decrease in fluorescence was observed, indicating a dose-dependent killing effect. As shown in Fig. 1a, OMS-A and OMS-B were very potent, with MICs lower than those of isoniazid, ethambutol, and SQ109. The MIC50 values for OMS-A and OMS-B were 57 nM and 117 nM, respectively.

We next assessed whether OMS-A and OMS-B could elicit antimicrobial responses against Mtb in macrophages. Treating Mtb-infected BMDMs with OMS-A (1, 10 μM) and OMS-B (1, 10 μM) inhibited the survival of intracellular Mtb in a dose-dependent manner after 3 days of infection (Fig. 1b). The number of colony forming units (CFUs) was profoundly reduced in BMDMs treated with OMS-A (10 μM) or OMS-B (10 μM), similar to those treated with isoniazid (0.5 μg/ml), compared with untreated cells (Fig. 1b, Fig. S1). Additionally, OMS-A (1, 10 μM) and OMS-B (1, 10 μM) exerted synergistic effects against Mtb (Fig. 1c). OMS-A and OMS-B had nearly equal inhibitory potential against Mtb; however, OMS-A exhibited slightly higher antimicrobial effects than did OMS-B (Fig. 1b). These data suggest that OMS-A and OMS-B induce antimicrobial responses against Mtb *in vitro* and in macrophages.

It was previously reported that the *M. marinum*-*D. melanogaster* infection system is an alternative model host for evaluating *Mycobacterium* infections^18^. Therefore, we evaluated whether this infection model could be used to effectively assess the antimicrobial effects of OMS-A *in vivo*. As shown in Fig. 1d, flies challenged with *M. marinum* died within ~20 days (500 CFU/50 nL), consistent with previous reports by Kim *et al*.^19^. We then monitored the survival of flies treated with OMS-A (1, 10 μM), which exhibited a significant decrease in lethality compared with those treated with the solvent control. Antibiotic control flies, which received food containing amikacin (1 μg/ml), showed comparable survival to those treated with OMS-A after *M. marinum* injection (Fig. 1d). In addition, the viable bacterial counts in surviving flies infected with *M. marinum* were monitored in the control and OMS-treated groups. The *in vivo* bacterial counts were consistently higher in control flies compared with flies treated with OMS-A 12 days after infection with *M. marinum* (n = 20 per group; Fig. 1e). These findings suggest that OMS-A exhibits *in vivo* antimicrobial activities against mycobacteria.

### OMS-A and OMS-B increase autophagy activation and autophagic flux in murine macrophages

**Figure 2:**
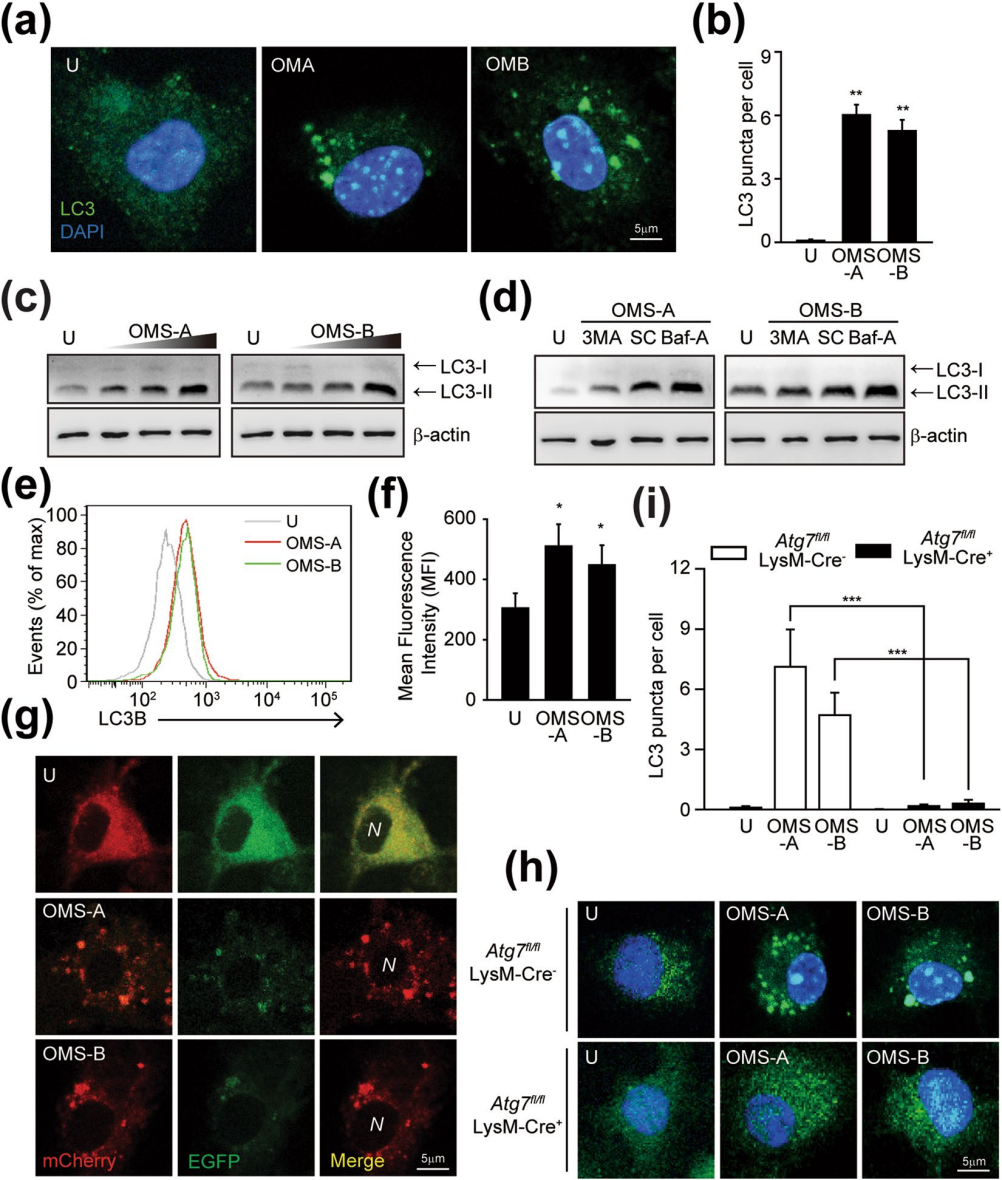
OMS induces autophagy activation in macrophages. (**a** and **b**) The Alexa 488-conjugated LC3 (green) and DAPI (blue) were detected by confocal microscopy at 24 h of OMS-A (10 μM; OMA) or OMS-B (10 μM; OMB) in BMDMs. (**a**) Representative of LC3 images. Scale bar, 5 μm. (**b**) Quantitative analysis of LC3 puncta per cell. Representative confocal microscopic images from three independent samples are shown, with each experiment including at least 100 cells scored from 6 random fields. (**c**) BMDMs were treated with OMS-A (1, 5, 10 μM) or OMS-B (1, 5, 10 μM) for 24 h. (**d**) BMDMs were pretreated with 3-MA (10 μM, for 2 h), or bafilomycin A1 (100 nM, for 1 h), and then treated with OMS-A or OMS-B for 24 h. (**c** and **d**) Cell lysates were subjected to immunoblot analysis of LC3 and Actin. The entire blots are shown in Supplementary Fig. 6. (**e** and **f**) Flow cytometric analysis of LC3B expression at 24 h of OMS-A or OMS-B treatment. (**f**) Quantitative analysis of LC3B expression. (**g**) BMDMs were transduced with retrovirus expressing a tandem-tagged mCherry-EGFP-LC3B and then treated with OMS-A or OMS-B for 24 h. Cells were tandem LC3B plasmid was detected by confocal microscopy. Scale bar, 5 μm. (**h**,**i**) Atg7^fl/fl^ LysM-Cre^−^ and Atg7^fl/fl^ LysM-Cre^+^ BMDMs were treated with OMS-A (10 μM) and OMS-B (10 μM) for 24 h. The Alexa 488-conjugated LC3 (green) and DAPI (blue) were detected by confocal analysis. (**h**) Representative of LC3 images. Scale bar, 5 μm. (**i**) Quantitative analysis of LC3 puncta per cell. Representative confocal microscopic images from three independent samples are shown, with each experiment including at least 100 cells scored from 6 random fields. Data shown are from one representative of at least three independent experiments (means ± SD of triplicates [**b**,**f**,**i**] samples). *p < 0.05, **p < 0.01, ***p < 0.001, compared with untreated control or *Atg7* ^fl/fl^ LysM-Cre^−^ (**b**,**e**). U, untreated; Baf-A, bafilomycin A1; SC, solvent control; N; nucleus.

We previously showed that treatment with antibiotics against Mtb infection resulted in autophagy, which is required for antimicrobial effects in the host^19^. Recent studies demonstrated that the important immunosuppressive agent cyclosporine A, a cyclic peptide, induces autophagic cell death in canine lens epithelial cells^20^. Thus, we assessed whether OMS-A or OMS-B enhances the activation of autophagy in BMDMs. Treatment with OMS-A (10 μM) or OMS-B (10 μM) caused no hazardous effects in BMDMs (data not shown). We next assessed LC3 puncta formation and lipidation, which are well-known indicators of autophagy induction^21^, in BMDMs treated with OMS-A (10 μM) or OMS-B (10 μM). As shown in Fig. 2a, fluorescent staining of LC3 puncta was increased in BMDMs treated with OMS-A (10 μM) or OMS-B (10 μM) for 24 h. Quantitative analysis showed that LC3 puncta formation was significantly increased by OMS-A and OMS-B treatment (Fig. 2b). It was noted that OMS-A and OMS-B had comparable effects on autophagy activation in BMDMs, similar to those observed in BMDMs treated with rapamycin (200 nM) or Torin1 (10 μM), the known autophagy activators^4, 22^ (Fig. S2).

Western blotting of cell lysates showed that OMS-A (1, 5, 10 μM) or OMS-B (1, 5, 10 μM) treatment induced LC3 lipidation in a dose-dependent manner, as indicated by an increase in the autophagosomal membrane-associated LC3-II fraction (Fig. 2c; Fig. S3a,b). In addition, the OMS-A or OMS-B-induced LC3-II fractions were significantly decreased by pretreatment with 3-methyladenine (3-MA; 10 μM), an inhibitor of autophagy, and increased by pretreatment with bafilomycin A1 (100 nM), a vacuolar H^+^-ATPase inhibitor (Fig. 2d). We previously used flow cytometric analysis using anti-LC3B-specific antibodies to assess activation of autophagy^19^. In the current study, flow cytometry showed a significant increase in LC3B levels in OMS-A (10 μM) or OMS-B (10 μM)-treated BMDMs (Fig. 2e,f). We further assessed the ability of OMS-A or OMS-B to induce autophagic flux^21^ in macrophages using a retroviral vector containing mCherry-enhanced green fluorescent protein (EGFP)-LC3B. Cells treated with OMS-A (10 μM) or OMS-B (10 μM) exhibited an increase in red puncta, suggesting that mCherry-EGFP-LC3B was delivered to lysosomes following OMS-A or OMS-B treatment (Fig. 2g).

We then examined the effects of autophagy in OMS-induced LC3 punctate formation. To examine this, BMDMs from *Atg7* ^fl*/*fl^LysM-Cre^+^ (*Atg7* KO) mice and their *Atg*7^+^ littermates control (*Atg7* wildtype; *Atg7* WT) were treated with OMS-A (10 μM) or OMS-B (10 μM), and LC3 punctate formation was compared. As shown in Fig. 2h and i, the OMS-A or OMS-B-induced increase in LC3-positive autophagosome formation in *Atg7* WT BMDMs was significantly decreased in *Atg7* KO BMDMs. Together, these data suggest that OMS-A or OMS-B treatment enhances autophagy activation and autophagic flux in BMDMs.

### OMS-A and OMS-B enhance Mtb phagosome maturation in BMDMs

**Figure 3:**
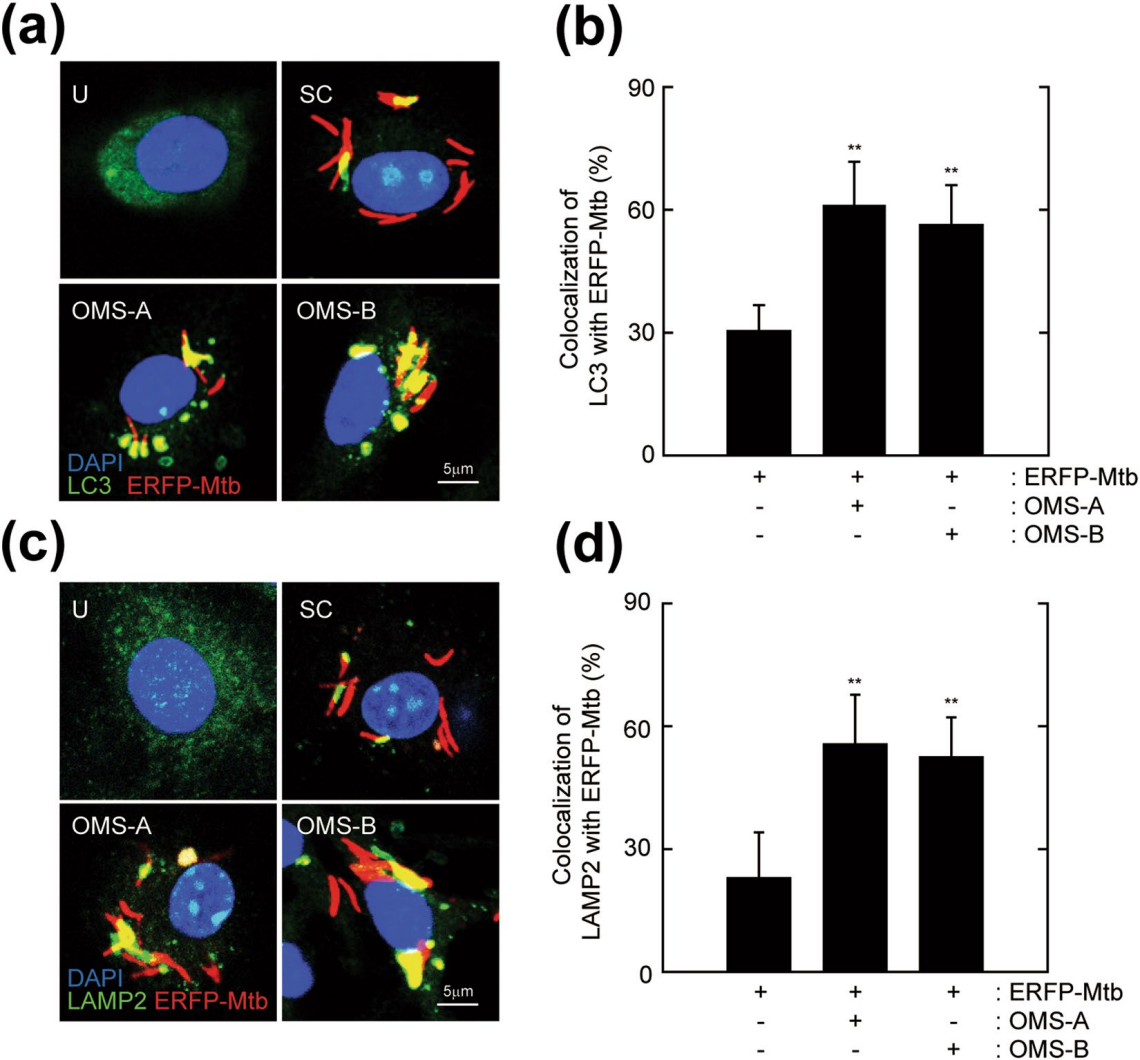
OMS-A and OMS-B activate phagosomal maturation against Mtb in macrophages. (**a**–**d**) BMDMs were infected with ERFP-Mtb (moi = 10) for 4 h and then treated with OMS-A (10 μM) or OMS-B (10 μM) for 24 h. (**a**) Mtb-ERFP (red), Alexa 488-conjugated-LC3 (green), and DAPI (blue) were detected by confocal analysis. Scale bar, 5 µm. (**b**) Quantitative analysis of Mtb-ERFP and LC3 colocalization per cell. (**c**) Mtb-ERFP (red), Alexa 488-conjuated LAMP2 (green), and DAPI (blue) were detected by confocal microscopy. Scale bar, 5 µm. (**d**) Quantitative analysis of Mtb and LAMP2 colocalization per cell. Representative confocal microscopic images from three independent samples are shown, with each experiment including at least 100 cells scored from 6 random fields (**a**,**c**). Data shown are from one representative of at least three independent experiments (means ± SD of triplicates [b, d] samples). **p < 0.01, compared with ERFP-Mtb-infected control (**b**,**d**). U, uninfected/untreated; SC, solvent control.

Mtb is a highly adapted pathogen that arrests the maturation of phagosomes into phagolysosomes^23^. Recent studies also showed that the Mtb virulent strain H37Rv significantly inhibited autophagic flux in macrophages^24^. The activation of antibacterial autophagy leads to mycobacterial phagosomal maturation, thus reducing the bacterial burden in macrophages^25^. To further evaluate OMS-A (10 μM) or OMS-B (10 μM) treatment-induced phagosome maturation, BMDMs were infected with enhanced red fluorescent protein (ERFP)-Mtb and then treated with OMS-A (10 μM) or OMS-B (10 μM). As shown in Fig. 3a–d, treating Mtb-infected BMDMs with OMS-A or OMS-B increased the co-localization of LC3, an autophagosomal marker, with Mtb phagosomes (Fig. 3a,b). Next, the co-localization of Mtb phagosomes with lysosomes was assessed in Mtb-infected BMDMs after OMS-A (10 μM) or OMS-B treatment (10 μM). Treating Mtb-infected BMDMs with OMS-A or OMS-B significantly enhanced the co-localization of Mtb phagosomes with LAMP2 (Fig. 3c,d).

In addition, we infected *Atg7* WT and *Atg7* KO BMDMs with ERFP-Mtb in the presence or absence of OMS-A or OMS-B. Notably, the LC3 punctate formation in Mtb-infected/OMS-A/OMS-B (10 μM) treated *Atg7* WT BMDMs was markedly decreased in *Atg7* KO BMDMs (Fig. S4a). In addition, the co-localization of bacterial phagosomes with the autophagosomal marker LC3 was significantly decreased in *Atg7* KO BMDMs compared to *Atg7* WT BMDMs (Fig. S4a,b). Collectively, these data indicate that OMS-A and OMS-B induce the phagosomal maturation of Mtb through autophagy activation in BMDMs.

### OMS-A and OMS-B activate the AMPK pathway, which is required for Mtb phagosomal maturation and antimicrobial responses in macrophages

**Figure 4:**
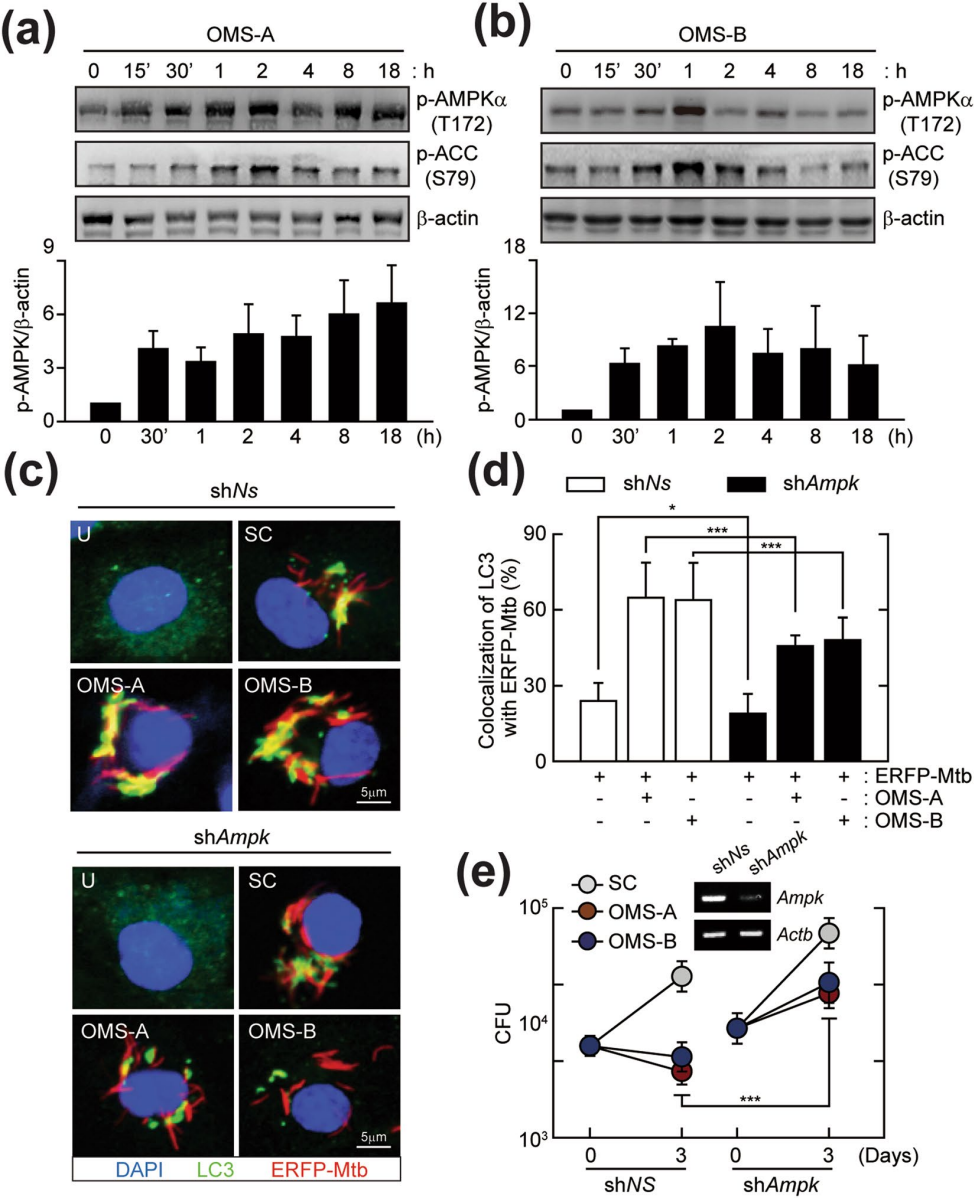
AMPK activation is required for OMS-induced phagosomal maturation and antimicrobial responses. (**a** and **b**) BMDMs were treated with OMS-A (10 μM; for **a**) or OMS-B (10 μM; for **b**) for indicated times (0–18 h). The cell lysates were subjected to immunoblot analysis of p-AMPKα, p-ACC, and Actin. The entire blots are shown in Supplementary Fig. 7. The densitometric values for p-AMPKα were normalized to Actin (*bottom*). (**c** and **d**) BMDMs were transduced with non-specific shRNA (sh*NS*) or *Ampk*-specific shRNA (sh*Ampk*)-expressing lentivirus for 48 h and then infected with ERFP-Mtb (moi = 10) for 4 h, followed by treatment with OMS-A or OMS-B for 24 h. (**c** and **d**) Mtb-ERFP (red), Alexa 488-conjugated-LC3 (green), and DAPI (blue) were detected by confocal analysis. Scale bar, 5 µm. (**c**) Representative confocal microscopic images from three independent samples are shown. (**d**) Quantitative analysis of cells showing the colocalization between LC3 and Mtb-ERFP. For each experiment, at least 100 cells were scored from 6 random fields. (**e**) After 3 days of infection, intracellular bacterial loads were determined by CFU assay. (inset) RT-PCR analysis of *Ampk* mRNA expression of transduction efficiency. Data shown are from one representative of at least three independent experiments (means ± SD of triplicates [**a**,**b** *bottom*; **d**] samples]). *p < 0.05, ***p < 0.001, compared with sh*NS*. U, uninfected/untreated; SC, solvent control.

Our previous studies showed that activation of AMPK contributes to autophagy activation and antimicrobial responses against Mtb^12^. We thus examined whether OMS-A or OMS-B activates AMPK by measuring the phosphorylation of Thr172 of the catalytic α-subunit of AMPK^26^. As shown in Fig. 4a,b, OMS-A and OMS-B induced the phosphorylation of AMPK at Thr172, as well as its downstream target acetyl-CoA carboxylase (ACC), in a time-dependent manner. Treating BMDMs with OMS-A (10 μM) or OMS-B (10 μM) rapidly increased AMPK phosphorylation within 0.5–1 h after stimulation. The AMPK phosphorylation levels were then slightly decreased in BMDMs after OMS-B stimulation, however, they were sustained in BMDMs in response to OMS-A and OMS-B at later time points (Fig. 4a,b).

To further define the role of AMPK in enhancing phagosome maturation, BMDMs were transduced with short hairpin RNAs (shRNA) lentiviral vectors that specifically target *Ampkα* (sh*Ampk*). The efficiency of *Ampkα* silencing was confirmed 48 h after transduction; the mRNA levels of *AMPK* were reduced compared with those in cells transduced with lentiviruses expressing nonspecific shRNA (sh*NS*). To examine the effects of *Ampkα* silencing on the colocalization of Mtb phagosomes and LC3 autophagosomes, BMDMs were transduced with sh*Ampk* or sh*NS*, infected with ERFP-Mtb, and then treated with or without OMS-A (10 μM) or OMS-B (10 μM). The colocalization of LC3 autophagosomes and Mtb phagosomes was markedly increased in sh*NS*-transduced BMDMs by treatment with OMS-A (10 μM) or OMS-B (10 μM). However, there was no significant difference between the effects of OMS-A and OMS-B on the co-localization of phagosomes and autophagosomes. In addition, silencing *Ampkα* dramatically attenuated OMS-induced colocalization of autophagosomes and Mtb phagosomes in BMDMs compared with those under the control conditions (Fig. 4c,d). Next, the ability of AMPK to promote the intracellular killing activities induced by OMS-A or OMS-B was examined. As shown in Fig. 4e, the inhibitory effects of OMS-A and OMS-B against Mtb in shNS-transduced macrophages were significantly counteracted by sh*Ampk*. These findings suggest that AMPK is the major signaling pathway mediating OMS-A and OMS-B-induced Mtb phagosome maturation and antimicrobial responses in macrophages.

### OMS-A and OMS-B activate autophagy to enhance antimicrobial responses against Mtb ***in vitro*** and ***in vivo***

**Figure 5:**
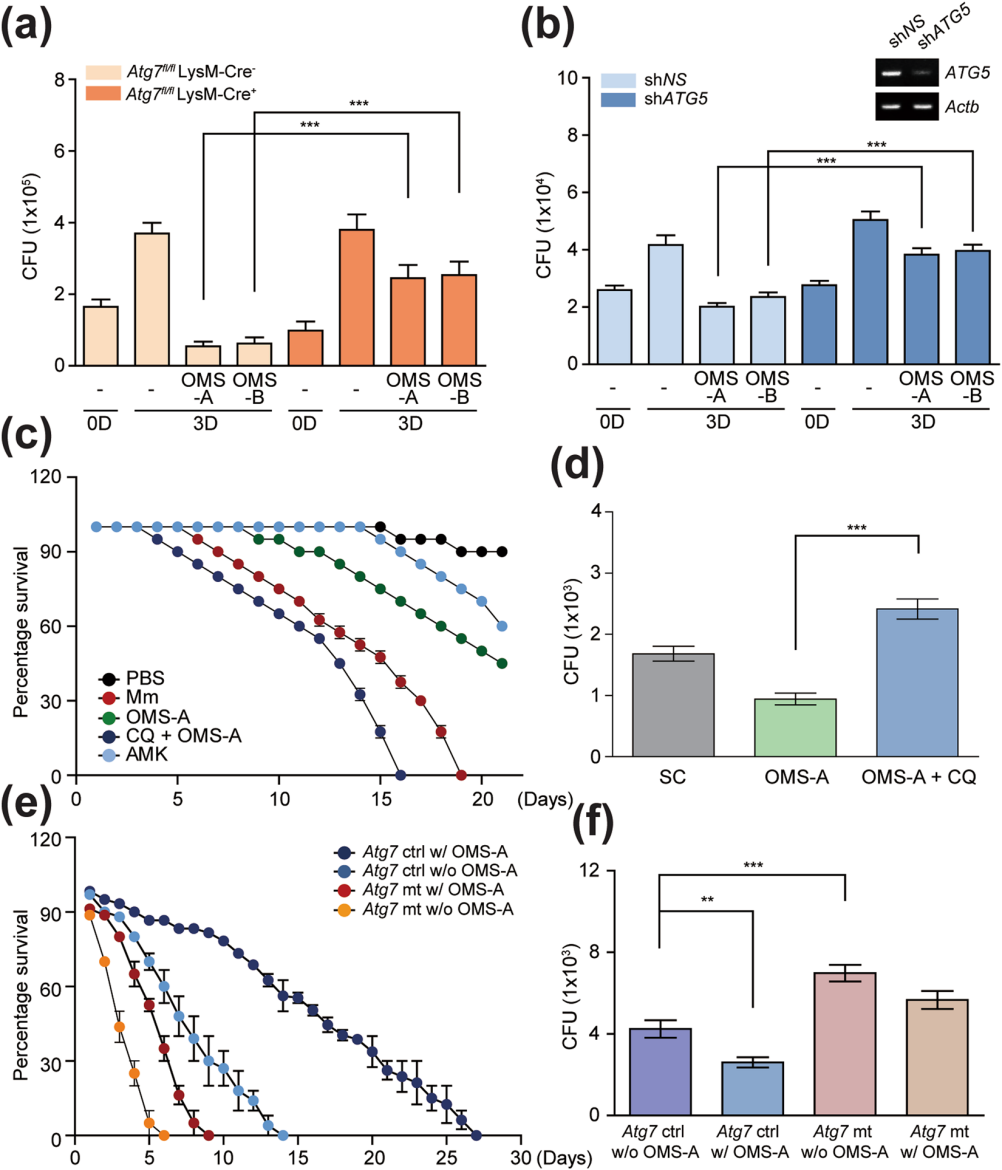
OMS-mediated autophagy activation is essentially required for antimicrobial responses against mycobacterial infection *in vitro* and *in vivo*. (**a**) *Atg7* ^fl/fl^ LysM-Cre^−^ and *Atg7* ^fl/fl^ LysM-Cre^+^ BMDMs were infected with Mtb (moi = 10) for 4 h and then treated with OMS-A (10 μM) and OMS-B (10 μM). (**b**) Human MDMs were transduced with non-specific shRNA (sh*NS*) or *ATG5*-specific shRNA (sh*ATG5*)-expressing lentivirus for 48 h and then infected with Mtb and then treated with OMS-A and OMS-B. (inset) RT-PCR analysis of *ATG5* mRNA expression of transduction efficiency. (**a** and **b**) CFU assay. The intracellular bacterial loads were determined at 3 days after infection. (**c** and **d**) W^1118^ flies were infected with *M. marium* (CFU = 500), followed by incubation with or without OMS-A (10 μM) in the presence or absence of chloroquine (1 μM). Positive control group was treated with AMK (1 μg/ml). (**e**) Live flies were counted at 24 h intervals (n = 40). The error bars indicate 95% confidence intervals. Log-rank analysis of the survival curves indicated that the susceptibility to *M. marinum* was significantly increased in the group of OMS-A with chloroquine, when compared with that of OMS-A only (***p < 0.001). (**f**) Each group (n = 20) of flies was harvested at 7 days, homogenized, and quantified by CFU assay. (**e** and **f**) *Atg7* control and *Atg7* mutant flies were infected with *M. marium* (CFU = 500), followed by incubation with or without OMS-A (10 μM). (**e**) Flies were counted at 24 h intervals (n = 50). The error bars indicate 95% confidence intervals. Log-rank analysis of the survival curves showed that the survival rates of *Atg7* control flies were significantly increased by OMS-A treatment (***p < 0.001), whereas these were not significant in *Atg7* mutant flies with the same treatment. (**f**) Each group (n = 20) of flies was harvested at 3 days, homogenized, and quantified by CFU assay. All data represent the means ± SD of triplicates from each sample. **p < 0.01, ***p < 0.001, compared with control conditions (**a**,**b**,**d**,**f**). SC, solvent control; AMK, amikacin; CQ, chloroquine.

To further examine the effects of autophagy in the antimicrobial responses by OMS, we performed additional cfu experiments using macrophages from *Atg7* KO mice. BMDMs from *Atg7* WT and *Atg7* KO were infected with Mtb, followed by treatment with or without OMS-A (10 μM) or OMS-B (10 μM). As shown in Fig. 5a, *Atg7* WT macrophages showed significantly increased killing effects against Mtb, whereas this was significantly inhibited in *Atg7* KO BMDMs following treatment with OMS-A (10 μM) or OMS-B (10 μM). It was also noted that OMS-A- or OMS-B-induced antimicrobial effects were substantially higher than those induced by another autophagy activators, such as rapamycin (100 nM) or Torin1 (10 μM) (data not shown). We next examined whether h*ATG5* is required for the OMS-mediated antimicrobial response against intracellular Mtb by transducing human monocyte-derived macrophages (MDMs) with shRNA against h*ATG5* (sh*ATG5)* and determining whether knockdown of *hATG5* affected the intracellular survival of Mtb in human MDM. Silencing of *ATG5* significantly counteracted the intracellular killing effects of OMS-A and OMS-B against Mtb in human MDMs (Fig. 5b; sh*NS* vs. sh*ATG5* under OMS-A (10 μM)- or OMS-B (10 μM)-treated conditions; *P* < 0.001, for both; moi = 10). These data indicate that OMS-mediated autophagy enhances the antimicrobial response in murine and human macrophages.

We further examined whether OMS-mediated antimicrobial effects were decreased in flies administered chloroquine, an inhibitor of the autophagy pathway^27^. We thus injected flies with *M. marinum* and treated them with OMS-A (10 μM) in the presence or absence of chloroquine (1 μM). We then monitored fly survival for 20 days. As shown in Fig. 5c, the flies treated with chloroquine exhibited increased lethality to *M. marinum* challenge compared to control flies, which were administered food containing OMS-A (10 μM) only. Consistent with these findings, viable bacterial counts in surviving flies were significantly increased by chloroquine administration relative to those in control flies treated with OMS-A only (after 7 days; *n* = 20 per group; Fig. 5d).

We then injected control and *Atg7* mutant flies with *M. marinum* in the presence or absence of OMS-A (10 μM) and monitored survival for 27 days. As shown in Fig. 5e, the *Atg7* mutant flies increased lethality to *M. marinum* challenge when compared to control flies. In addition, administration of control flies with food containing OMS-A (10 μM) led to a significant increase of survival after *M. marinum* injection. However, OMS-A-mediated protective effects were not significant in *Atg7*-mutant flies after *M. marinum* infection (Fig. 5e). Furthermore, viable bacterial counts of *M. marinum* were significantly higher in *atg7* mutant flies than those in control flies infected with *M. marinum* (after 3 days; *n* = 20 per group; Fig. 5f). OMS-A treatment significantly decreased the bacterial loads in control flies, but did not decrease the viable bacteria in *Atg7* mutant flies to the levels observed in control flies (Fig. 5f). These findings suggest that host autophagy activation is required for the OMS-induced antimicrobial effects against mycobacterial infection *in vitro* and *in vivo*.

### OMS-A and OMS-B attenuate Mtb-induced inflammatory responses by activating AMPK in macrophages

**Figure 6:**
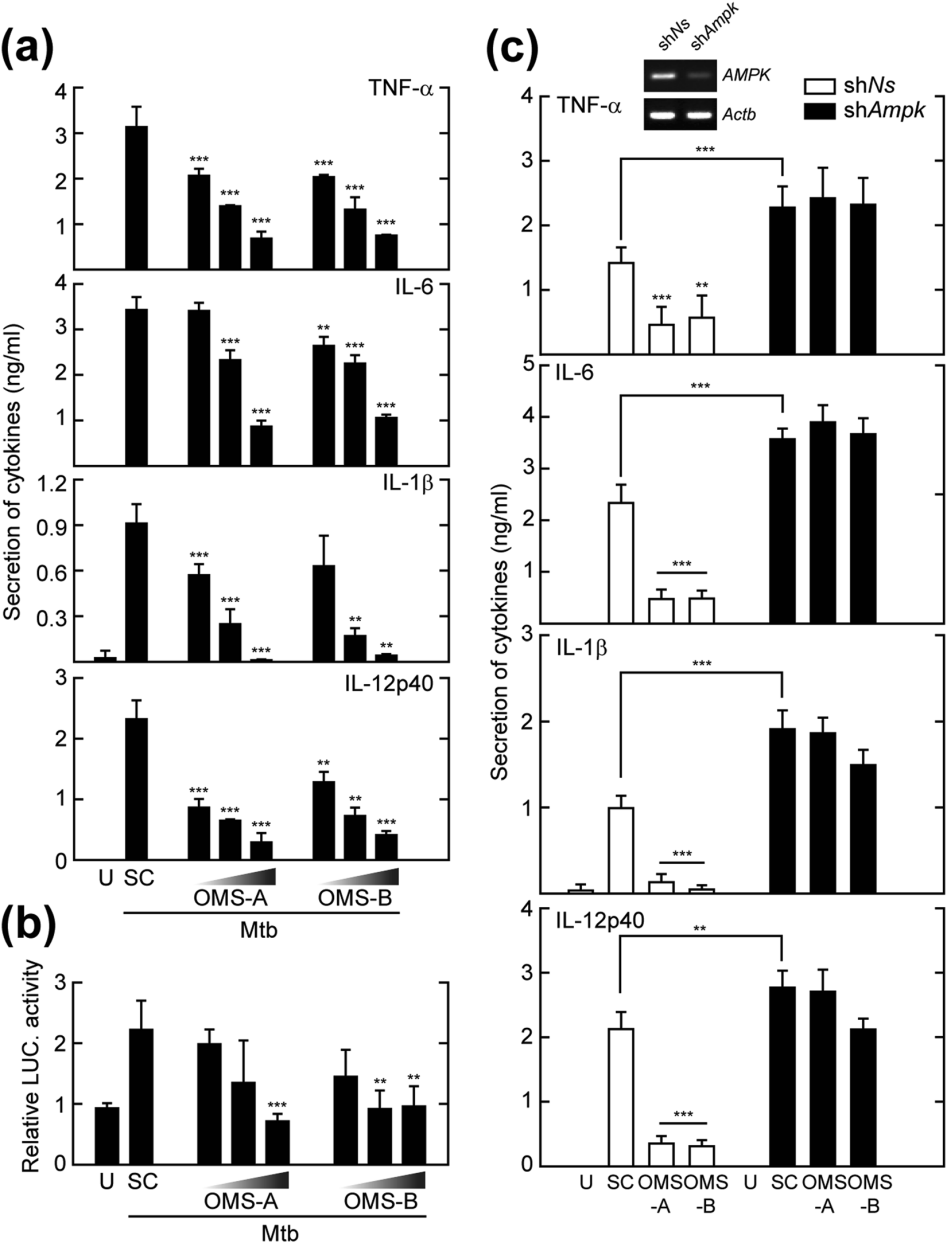
OMS inhibits Mtb-induced inflammatory responses through AMPK activation. (**a**) BMDMs were infected with Mtb (moi = 10) for 4 h and then treated with OMS-A (1, 5, 10 μM) or OMS-B (1, 5, 10 μM) for 24 h. (**b**) BMDMs were transduced with adenoviral NF-ĸB-luciferase reporter plasmid for 36 h, and infected with Mtb and then treated with OMS-A or OMS-B for 6 h. The cells were harvested and NF-ĸB luciferase reporter activity was determined. (**c**) BMDMs were transduced with non-specific shRNA (sh*NS*) or *Ampk*-specific shRNA (sh*Ampk*)-expressing lentivirus for 48 h and then infected with Mtb, followed by treatment with OMS-A or OMS-B for 24 h. (**a** and **c**) The supernatants were harvested and subjected of ELISA analysis of TNF-α, IL-6, IL-1β, and IL-12p40 production. All data represent the means ± SD of triplicates from each sample. **p < 0.01, ***p < 0.001, compared with SC (**a**–**c**). U, uninfected/untreated; SC, solvent control; ns, no significant.

Accumulating evidence has shown that AMPK signaling is required for anti-inflammatory responses^28^. However, it is unclear whether AMPK activation is essential for the inhibition of Mtb-induced inflammatory responses. We first examined cytokine production in Mtb-infected BMDMs after treatment with OMS-A (1, 5, 10 μM) or OMS-B (1, 5, 10 μM). As shown in Fig. 6a, Mtb-induced production of proinflammatory cytokines, such as tumor necrosis factor (TNF)-α, interleukin (IL)−6, IL-1β, and IL-12 p40, was dose-dependently inhibited by treatment with OMS-A (1, 5, 10 μM) or OMS-B (1, 5, 10 μM) in BMDMs. In addition, Mtb-induced nuclear factor kappa B (NF-κB) reporter gene activity was abrogated by OMS-A (1, 5, 10 μM) or OMS-B (1, 5, 10 μM) treatment in a dose-dependent manner (Fig. 6b).

Finally, we examined whether AMPK is involved in the attenuation of proinflammatory cytokine production in BMDMs. BMDMs were transduced with sh*Ampk* or sh*NS*, infected with Mtb, and then treated with OMS-A or OMS-B. As shown in Fig. 6c, the OMS-mediated inhibition of proinflammatory cytokine production was nearly completely counteracted by sh*Ampk* in BMDMs. Collectively, these data suggest that OMS-induced AMPK activation is required for suppression of Mtb-induced inflammatory responses in BMDMs.

## Discussion

Much of the recent focus regarding therapeutic strategies against Mtb has been on the development of host-directed therapies that enhance antimicrobial mechanisms, such as autophagy inducers, cathelicidin inducers, and anti-inflammatory agents^29, 30^. Novel functions and mechanisms by which various autophagy inducers enhance antibacterial autophagy and antimicrobial responses have been reported^31, 32^. Early studies showed that interferon (IFN)-γ^5^, vitamin D^10, 33, 34^, and both treatments synergize the elimination of Mtb via autophagy^10^. Various innate signals and pathogen- and damage-associated molecular patterns enhance the Mtb-induced inhibition of intracellular growth by activating autophagic machinery, interacting with innate signaling pathways, and delivering antimicrobial peptides to phagosomes^10, 33, 35–37^. Here, we demonstrated that the newly identified cyclic peptides OMS-A and OMS-B activate autophagy via the AMPK pathway, to promote antimicrobial effects against Mtb. In addition, OMS-induced autophagy leads to antimicrobial responses to Mtb in macrophages, as shown by intracellular survival assays.

Since issues regarding multidrug-resistant Mtb are increasing worldwide, there is an urgent need to develop new antitubercular drugs. The current data suggest that OMS-A and OMS-B exhibit at least three attractive anti-tubercular drug activities: 1) direct antimicrobial killing activity against Mtb, 2) direct activation of autophagy, which was essential for antimicrobial effects, in host cells, and 3) inhibition of Mtb immunopathology. REMAs revealed that OMS-A and OMS-B have excellent MICs and MBCs against Mtb, comparable to those of isoniazid and ethambutol, which are the antibiotics currently used against human tuberculosis. Our previous studies revealed that OMS-A and OMS-B are cyclic peptides that harbor activity against several bacteria, as well as exhibit an inhibitory effect on cancer cell proliferation^16^. Recently, chemical investigations identified several cyclic peptides as promising anti-Mtb agents due to strong antitubercular activities *in vitro* ^38^. In addition, a high-throughput screening study revealed that ecumicin is an excellent drug candidate with activity against multidrug-resistant (MDR) and extensively drug-resistant (XDR) Mtb strains^39^. Lassomycin, a basic and ribosomally encoded cyclic peptide, also exhibits bactericidal activity against mycobacteria^40^. The mycobacterial ClpC1 ATPase complex was identified as a drug target for both ecumicin and lassomycin^39, 40^. Although we did not try to identify the targets of OMS-A and OMS-B, we speculate that the same ClpC1 ATPase complex could be a potential target. Indeed, significant effort in developing new cyclic antimicrobial peptides has been devoted to designing analogs of drugs that reduce cytotoxic side effects but promote antitubercular and antibacterial properties using conformational analyses based on structure-activity relationships^41^. Importantly, we did not observe any toxic effects on host macrophages and flies *in vivo* treated with OMS-A and OMS-B, at least at the doses and time periods evaluated. Nevertheless, further studies are needed to develop a large scale of OMS and design safe and effective chemical analogs for future clinical trials.

Both OMS-A and OMS-B activated autophagy, which is an essential part of the antimicrobial defense against Mtb. Our data demonstrated that OMS-A and OMS-B-induced autophagy is essential for phagosomal maturation, and for antimicrobial effects against Mtb infection, both *in vitro* and in flies. The findings of this study were in partial agreement with our previous studies in showing that isoniazid and pyrazinamide, two antibiotics used to treat tuberculosis in humans, activate the antibacterial autophagy required for successful antimicrobial activity during mycobacterial infection^19^. In a recent report, thiostrepton, a thiopeptide antibiotic possessing a quinaldic acid moiety, was used successfully in the treatment of *M. marinum*, through ER-stress-mediated autophagy^42^. In addition, a search for active compounds to enhance interferon-gamma responses in macrophages identified several such compounds within the flavagline (rocaglate) family, which effectively induce autophagy to combat intracellular mycobacteria and inhibit pathologic inflammation during infection^43^.

These OMS-A and OMS-B activities were related to their ability to activate AMPK in macrophages. AMPK is an energy sensor that regulates energy balance at a cell-autonomous level in living organisms^11^. Much attention has been given to promising adjunctive host-directed therapeutic candidates that affect cell signaling pathways associated mainly with immunometabolism (i.e., the core pathways intersecting immunologic and metabolic responses)^44^. Understanding the mechanisms that regulate AMPK and mammalian target of rapamycin signaling will likely facilitate significant advances in the development of adjunctive options for several unmet needs regarding tuberculosis, including new drugs against MDR and XDR strains, shortening the chemotherapeutic treatment duration, and targeting various clinical stages, even in combination with human immunodeficiency viral infection^44, 45^. Recent studies demonstrated that AMPK plays a key role in antimicrobial autophagy against mycobacteria^44^ and that several AMPK activators have the potential to activate autophagy and intracellular killing in response to Mtb^12, 46^. In addition, the widely used anti-diabetes drug metformin, which exhibits AMPK-activating activity, was identified as an excellent adjunct antituberculous therapy^47^. The current data demonstrate that AMPK activation by OMS-A and OMS-B could impact maturation. These studies collectively suggest that activation of AMPK activation in host cells enhances the cell-autonomous and *in vivo* killing effects against mycobacteria.

NF-κB activation and inflammatory responses during Mtb infections appear to be a double-edged sword, i.e., a defense reaction against microbial insults and immunopathologic responses in tuberculosis. Indeed, the design of immunomodulatory agents has been focused on host-directed therapeutics that facilitates protective immune responses while simultaneously reducing deleterious inflammatory responses^45^. The current data revealed that OMS-induced AMPK activation inhibited inflammatory responses in macrophages induced by Mtb infection. Activation of the AMPK signaling pathway promotes anti-inflammatory and anti-cancer effects by inducing oxidative metabolism^28^. AMPK activators such as metformin and salicylate suppress inflammatory responses in various cell types by modulating NF-κB or other signaling pathways^28, 48, 49^. In addition, autophagy controls excessive inflammatory responses and type I interferon activation, particularly during microbial infections^50^. Previous studies showed that autophagy modulates NF-κB signaling pathways during innate immune responses to attenuate exacerbated inflammation. It was reported that inhibition of NF-κB signaling in human macrophages increased apoptosis and autophagy, which subsequently decreased intracellular Mtb survival^51^. Previous studies revealed the mechanisms by which autophagy regulates NF-κB signaling. Specifically, hepatoma tumor cell-conditioned medium induced TLR2 signaling, which stimulated the degradation of NF-κBp65 by SQSTM1/p62-mediated selective autophagy^52^. Thus, OMS-induced anti-inflammatory responses may be the result of at least two mechanisms: AMPK-dependent- and autophagy-activating pathways.

Taken together, these results provide a novel mechanism underlying the antimicrobial effects of the novel cyclic peptides OMS-A and OMS-B, via AMPK-dependent autophagy activation, thus overcoming the inhibition of phagolysosomal fusion caused by pathogens. OMS-A and OMS-B induced a direct killing activity against Mtb and, interestingly, promoted host defenses; they did not only activate autophagy but also regulated pathologic inflammatory responses during Mtb infection. These efforts may contribute to the development of new therapeutic drugs and biologics by enhancing dual effects on both the bacteria and host immunity.

## Materials and Methods

### Cultivation and extraction of the actinobacterial strain SNJ042 to produce OMS-A and B

Isolation of OMS-A and OMS-B were performed as described previously^16^. The actinobacterial strain SNJ042 (99% identity with *Streptomyces cheonanensis* based on 16 S ribosomal DNA analysis) was isolated from a sand beach in Jeju Island, Republic of Korea, and was cultivated to produce OMS-A and OMS-B. It was incubated on solid YEME medium (10 g malt extract, 4 g yeast extract, 4 g glucose, and 18 g agar powder in 1 L sterilized 3.4% artificial seawater) at 25 °C to acquire spores. The spores were inoculated into 125 ml liquid A1/C medium (10 g starch, 4 g yeast extract, 2 g peptone, and 1 g CaCO_3_ in 1 L sterilized 2.4% artificial seawater) in a 500 ml Erlenmeyer flask. The liquid culture was incubated at 30 °C with shaking at 200 rpm. After 2 days, 10 ml of the liquid culture were transferred to 1 L liquid A1/C medium in a 2.8 L Fernbach flask and further cultivated on a rotary shaker at 30 °C and 180 rpm (108 × 1 L, total culture volume 108 L). After 7 day cultivation, 1.5 L ethyl acetate (EtOAc) were added to each 1 L bacterial culture and extracted twice using a separation funnel. The EtOAc layer was collected in an empty 2.8 L Fernbach flask, and anhydrous sodium sulfate was added to the organic extract to remove residual water. The extract in EtOAc was concentrated *in vacuo* to yield 12 g dry material.

### Determining the MIC by REMA

The REMA was performed as described previously to determine the MICs of OMS-A or OMS-B, isoniazid (INH; I3377), ethambutol (E4630), and SQ-109 (SML1309) (all from Sigma, St. Louis, MO, USA) against H37Rv^17^. Briefly, a 100 µl inoculum was used to inoculate each well of the plate, and two-fold serial dilutions of each test compound were prepared in 96-well plates in triplicate. An inoculum at an optical density 600 of 0.005 was prepared by diluting mid-log cultures and then added to each well. Growth controls containing no drug and a sterile control were also prepared in each experiment. Plates were incubated at 37 °C for 5 days, and 40 µL 0.025% resazurin (Sigma, R7017) solution were then added to each well. After an overnight incubation, the fluorescence of the resazurin metabolite resorufin was determined by excitation at 560 nm and emission at 590 nm using the Synergy H1 microplate reader (Biotechnology Inc, Dallas, TX, USA). The MIC50 (the MIC required to inhibit the growth of 50% of the organism) was determined using GraphPad Prism 5.0 software.

### Mycobacterial cultures

Mycobacterial cultures were performed as described previously^12^. Mtb H37Rv was provided by Dr. R.L. Friedmann (University of Arizona, Tucson, AZ, USA). Mycobacteria were grown in Middlebrook 7H9 medium (Difco, Detroit, MI, USA, 271310) supplemented with 10% oleic albumin dextrose catalase (OADC; BD Biosciences, Franklin Lakes, NJ, USA, 212240), 5% glycerol, and 0.05% Tween 80 (Sigma, P1754). The Mtb strains expressing ERFP were described previously^53^. The *E. coli* Mtb shuttle plasmid pMV262-RFP harboring ERFP under the control of the *HSP60* promoter was used. Mtb harboring the ERFP gene was cultivated in 7H9 medium supplemented with kanamycin (Sigma, 60615). For *D. melanogaster* infection, the *M. marinum* strain Aronson (ATCC 927, fish isolate) was cultured in 7H9 medium with OADC and 0.2% Tween 80 at 30 °C for 8 weeks in the dark without agitation. Single-cell suspensions of Mtb, Mtb-ERFP, and *M. marinum* were aliquoted and stored at −80 °C. Mid-logarithmic-phase bacteria (OD, 0.6) were used in all assays. The CFUs were enumerated on Middlebrook 7H10 agar (Difco, 262710).

### Mice and Cells

C57BL/6 mice aged 6–8 weeks with a WT background were purchased from Samtako Bio Korea (Gyeonggi-do, Korea). We generated with myeloid lineage cell-specific *Atg7*-deficient mice (*Atg7* ^fl/fl^LysM-Cre^+^) using the Cre/loxP recombination system, as described previously^54^. This study was approved by the Institutional Research and Ethics Committee at Chungnam National University School of Medicine (CNUH-01-A0008; Daejeon, Korea). All animal procedures were conducted in accordance with the guidelines of the Korean Food and Drug Administration. Mice were maintained under specific pathogen-free conditions.

BMDMs were isolated and differentiated as described previously^12^. BMDMs were cultured for 5 days in Dulbecco’s modified Eagle’s medium (DMEM; Lonza, 12–604 F) medium containing 25 ng/ml macrophage colony-stimulating factor (M-CSF; R&D Systems, 416-ML) and supplemented with 10% heat-inactivated fetal bovine serum (FBS; Lonza, BW14–503E) and penicillin-streptomycin-amphotericin B (Lonza, 17–745E).

Human MDMs were as described previously^19^. Briefly, peripheral blood mononuclear cells were isolated from healthy donors by density gradient centrifugation using Lymphoprep (Axis-Shield, Oslo, Norway). Human MDMs were cultured for 5 days in RPMI1640 (Lonza, 12–702 F) medium containing 10% pooled human serum and of 4 ng/ml human M-CSF (Sigma). Written infomed consent was obtained from all participants before enrolment in the study. The study was approved by the institutional review board of the Chungnam National University Hospital, and was done in accordance with the Declaration of Helsinki and good clinical practice guidelines.

### Reagents and antibodies (Abs)

3-MA (M9281), 4,6-diamidino-2-phenylindole dihydrochloride (DAPI, D9542), LC3 (L8918), dimethyl sulfoxide (D8418), and N’-(7-chloroquinolin-4-yl)-N,N-diethylpentane-1,4-diamine (chloroquine; C6628) were purchased from Sigma. Bafilomycin A1 (196000) was purchased from Calbiochem (San Diego, CA, USA). Alexa 488-conjugated anti-rabbit IgG (A17041) was purchased from Molecular Probes (Eugene, OR, USA). Actin (sc-1616) was purchased from Santa Cruz Biotechnology (Santa Cruz, CA, USA). Anti-phospho-ACC (3661) and anti-phospho-AMPKα (2535) Abs were purchased from Cell Signaling Technology (Beverly, MA, USA).

### Generation and transduction of small hairpin RNA (shRNA)

Lentivirus production was performed as described previously^33^. Briefly, the lentiviral construct vector pLKO.1 and three packaging plasmids (pRSVRev, pMD2.VSV-G, and pMDLg/pRRE) were purchased from Addgene. The pLKO.1-based target shRNA plasmids for m*Ampkα* (Santa Cruz Biotechnology, sc-29674-SH) or h*ATG5* (Santa Cruz Biotechnology, sc-41445-SH) were cotransfected into HEK293T cells using Lipofectamine 2000 (Invitrogen, 12566014, Carlsbad, CA, USA) for 72 h. Then, the lentivirus-containing supernatant was collected, filtered, and titrated. For lentivirus transduction, BMDMs or MDMs were infected with lentiviral vectors using 8 μg/mL polybrene (Sigma, 107689) for 48 h. Transduced cells were harvested, and the target gene knockdown efficiency was analyzed.

### Generation of tandem LC3B retrovirus

Tandem LC3B plasmid (mCherry-EGFP-LC3B) was performed as described previously^21^. To produce tandem LC3 retrovirus, human Phoenix amphotropic (ATCC, CRL-3213) cells were seeded at 70–80% confluence into a 6-well plate and co-transfected with 0.75 μg of the packaging plasmid pCL-Eco (Addgene, 12371), 0.25 μg of the envelope plasmid pDM2.G (Addgene, 12259) and 1 μg pBABE-puro Tandem LC3B plasmid using Lipofectamine 2000 for 6 h. Subsequently, the media containing tandem LC3B retrovirus were replaced with fresh media and cultured for 48 h. The tandem LC3B retrovirus was then harvested and filtered through a 0.45 μm syringe filter.

### CFU assay

To quantify intracellular bacteria, CFU assays were performed as described previously^12^. BMDMs were plated on 7H10 at a concentration of 2 × 10^5^ cells per well and infected with Mtb for 4 h. The cells were then washed with phosphate buffered saline (PBS) to remove extracellular bacteria and treated with OMS-A, OMS-B, or INH in medium for 3 days. Thereafter, the intracellular bacteria were harvested, and the lysates were diluted two-fold in PBS. Each sample was plated on 7H10 agar plates and incubated at 37 °C in a 0.5% CO_2_ incubator for 3 weeks.

### Western blotting and enzyme-linked immunosorbent assay (ELISA)

Western blotting and ELISA were performed as described previously^55^. For western blotting, cell lysates were denatured by boiling and separated on 12% acrylamide SDS-PAGE gels. Then, proteins were transferred to polyvinyl difluoride membranes (Millipore, Boston, MA, USA) and incubated with primary Abs (LC3, p-AMPKα, and p-ACC) diluted at a ratio of 1:1000. The bands were visualized using ECL (Millipore, Danvers, MA, USA) and exposure to chemiluminescence film (Fujifilm, Tokyo, Japan).

For ELISA, infected cell supernatants were used to measure mouse TNF-α (ELISA Max™ Standard Set; BioLegend, Inc., San Diego, CA, USA), IL-6 (BioLegend), IL-1β (BioLegend), and IL-12p40 (BD Pharmingen, San Diego, CA, USA) secretion according to the manufacturer’s instructions.

### RNA extraction and semi-quantitative RT-PCR

RNA extraction and semi-quantitative RT-PCR were performed as described previously^55^. Briefly, total RNA from cells was isolated using TRIzol reagent (Thermo Fisher Scientific, 15596–026) and used for synthesis of cDNA with Superscript II reverse transcriptase (Invitrogen). PCR reactions involved 30 cycles of annealing, extension, and denaturation the following primers were used: m*Ampk* forward 5′-GGTGTACGGAAGGCAAAATGGC-3′, reverse 5′-CAGGATTCTTCCTTCGTACACGC-3′; m*Actb* forward 5′-CATTGCTGACAGGATGCAGAAGG-3′, reverse 5′-TGCTGGAAGGTGGACAGTGAGG-3′, h*ATG5* forward 5′-GCAGATGGACAGTTGCACACAC-3′, reverse 5′-GAGGTGTTTCCAACATTGGCTCA-3′; and h*ACTB* forward 5′-CACCATTGGCAATGAGCGGTTC-3′, 5′-AGGTCTTTGCGGATGTCCACGT-3′. Reactions were run on a Veriti 96-Well Thermal Cycler (ThermoFisher). The PCR products were analyzed by electrophoresis on 1.5% agarose gel.

### Flow cytometry

Intracellular LC3B levels were analyzed as described previously^12^. Stimulated cells were washed with PBS, fixed with 4% paraformaldehyde for 10 min at room temperature (RT), and permeabilized using 0.25% Triton X-100 in PBS for 10 min at RT. Cells were stained with primary Abs against LC3B (2775 S; Cell Signaling, 1:100) for 1–2 h at 4 °C followed by anti-rabbit IgG secondary Abs (7074 S; Cell Signaling, 1:100) for 1 h on ice. After two washes with PBS, cells were fixed in 4% paraformaldehyde and assayed immediately. The cells were examined using a FACSCanto II flow cytometer (Becton Dickinson, San Jose, CA, USA). Data were collected from 10,000–30,000 cells and analyzed using FlowJo software (Tree Star, Ashland, OR, USA).

### Confocal microscopy of autophagy analysis

LC3 punctate and LAMP2 staining and quantification were performed as described previously^12^. After treatment, cells on coverslips were washed twice with PBS, fixed with 4% paraformaldehyde for 20 min, permeabilized with 0.25% Triton X-100 in PBS for 10 min, and immunostained with primary Abs (anti-LC3; PM036, MBL International, 1:400) or (anti-LAMP2; sc-5571, Santa Cruz, 1:400) for 2 h. Cells were washed to remove primary Abs and incubated with the fluorescently labeled secondary Ab (Alexa 488-conjugated anti-rabbit IgG; A17041, Molecular Probes, 1:400) for 2 h. Nuclei were stained by incubation with DAPI (D9564, Sigma) for 5 min. After mounting, fluorescence images were acquired using a confocal laser-scanning microscope (LSM 710, Zeiss, Thomwood, NY, USA).

LC3 punctate dot fluorescence intensity was measured using ImageJ analysis software. For colocalization analysis, the co-distribution of LC3 autophagosomes or LAMP2 phagolysosomes with bacterial phagosomes were quantified using the ImageJ analysis software. Each condition was assayed in triplicate, and at least 100 cells per well were counted.

### *M. marinum* infection and intracellular bacterial growth in *D. melanogaster*

The culture, *M. marinum* infection, and intracellular bacterial growth of W^1118^, *CG5335* ^*d30*^ */Atg7* ^*d14*^ (*Atg7* control), and *Atg7* ^*d77*^ */Atg7* ^*d14*^ (*Atg7* mutant) files were performed as described previously^12, 56^. For *M. marinum* infections, 3–5 days old male W^1118^, *Atg7* control, and *Atg7* mutant flies were anesthetized with CO_2_ and injected with 500 CFU *M. marinum* in 50 nl (total volume) PBS using an individually calibrated pulled-glass needle attached to a microinjector (IM-5B; Narishige, Japan). Then, *M. marinum*-infected flies were incubated with standard cornmeal medium, OMS-A (1, 10 μM), amikacin (1 μg/ml) or co-treated with chloroquine (1 μM) and OMS-A (10 μM) containing medium and maintained at 25 °C and 60% humidity. All flies were transferred to fresh vials at least twice per week.

To determine bacterial growth, *M. marinum*-infected live W^1118^, *Atg7* control, and *Atg7* mutant flies were incubated with various containing medium (OMS-A or co-treated with chloroquine and OMS-A) for indicated days (n = 20/group, three independent experiments). The homogenized *M. marinum*-infected flies were serially diluted and plated onto 7H10 agar. After 14 days, the number of colonies on each plate was counted.

### NF-κB luciferase reporter assay

NF-κB luciferase reporter assays were performed as described previously^55^. Briefly, BMDMs were transduced with adenoviruses harboring a NF-κB-luciferase reporter plasmid (Genetransfer Vector Core, Iowa, IA, USA) for 36 h. The cells were then infected with Mtb for 4 h and stimulated with OMS-A or OMS-B for 6 h. The cells were washed three times in PBS and lysed in luciferase lysis buffer (E1531, Promega, Madison, WI, USA). A luciferase assay system (E1501, Promega) was used according to the manufacturer’s instructions.

### Statistical analyses

All data obtained from independent experiments are presented as means ± SD. Group-wise post-hoc comparisons were performed using the Student’s *t-test* in GraphPad Prism 5.0. Differences were considered statistically significant if *P* < 0.05.

## Electronic supplementary material

Supplementary Information
