# Supplementary material for: Ohmyungsamycins promote antimicrobial responses through autophagy activation via AMP-activated protein kinase pathway
Source: Sci Rep. 2017 Jun 13;7:3431. doi: 10.1038/s41598-017-03477-3 (PMC5469788; doi:10.1038/s41598-017-03477-3)
Supplement: Supplementary file 1 — Supplementary Information [file 41598_2017_3477_MOESM1_ESM.doc]

**Supplementary information**

**Ohmyungsamycins promote antimicrobial responses through autophagy activation via AMP-activated protein kinase pathway**

Tae Sung Kim^1,2*^, Yern-Hyerk Shin^3*^, Hye-Mi Lee^1*^, Jin Kyung Kim^1,2^, Jin Ho Choe^1,2^,

Ji-Chan Jang^4^, Soohyun Um^3^, Hyo Sun Jin^1,2^, Masaaki Komatsu^5^, Guang-Ho Cha^6^,

Han-Jung Chae^7^, Dong-Chan Oh^3^ and Eun-Kyeong Jo^1,2^

^1^Department of Microbiology, ^2^Department of Medical Science, ^6^Department of Infection Biology, Chungnam National University School of Medicine, Daejeon 35015, South Korea, ^3^Natural Products Research Institute, College of Pharmacy, Seoul National University, Seoul 08826, South Korea. ^4^Molecular Mechanism of Antibiotics, Division of Life Science, Research Institute of Life Science, Gyeongsang National University, Jinju 52828, South Korea. ^5^Department of Biochemistry, Niigata University Graduate School of Medical and Dental Sciences, Niigata 9518510, Japan. ^7^Department of Pharmacology, Chonbuk National University Medical School, Jeonju 54907, South Korea.

^*^These authors contributed equally to this work

Address correspondence and reprint requests to

Dr. Dong-Chan Oh

Phone: 82-2-880-2491

e-mail: [dongchanoh@snu.ac.kr](mailto:dongchanoh@snu.ac.kr)

Dr. Eun-Kyeong Jo

Phone: 82-42-580-8243

e-mail: [hayoungj@cnu.ac.kr](mailto:hayoungj@cnu.ac.kr)

**Supplementary materials and methods**

**Chromatographic fractionation of OMS**

The dry extract was adsorbed on Celite and then loaded onto 20 g C_18_ resin for fractionation. Chromatographic fractionation was performed using a step gradient solvent system by applying a sequential mixture of methanol (MeOH) and water (200 ml each of 20%, 40%, 60%, 80%, and 100% MeOH/H_2_O). OMS-A and OMS-B were detected in the 80% and 100% MeOH/H_2_O fractions. The fractions were subjected to semi-preparative reversed-phase HPLC using a gradient solvent system (Kromasil C_18_ (2); 250 × 10 mm; flow rate, 2 ml/min; UV detector, 280 nm; 40–56% acetonitrile/water gradient over 20 min followed by a 60% acetonitrile/water isocratic system after 20 min) for purification of OMS-A and OMS-B. OMS-A (70 mg) and OMS-B (20 mg) were eluted as pure compounds with retention times of 32 and 34 min, respectively. The purities of OMS-A and OMS-B were further confirmed by LC/MS (reversed-phase HPLC gradient system: Phenomenex C_18_ (2), 100 × 4.6 mm; flow rate, 0.7 ml/min; UV detector, 280 nm; 10-100% acetonitrile/water with 0.1% formic acid over 20 min) (Fig. S5).

**Reagents and Abs**

Rapamycin (553211) was purchased from Calbiochem.Torin1 (4247) was purchased from Tocris Bioscience (Avonmouth, Bristol, UK). Anti-phospho-mTOR (2971) was purchased from Cell Signaling Technology.

**
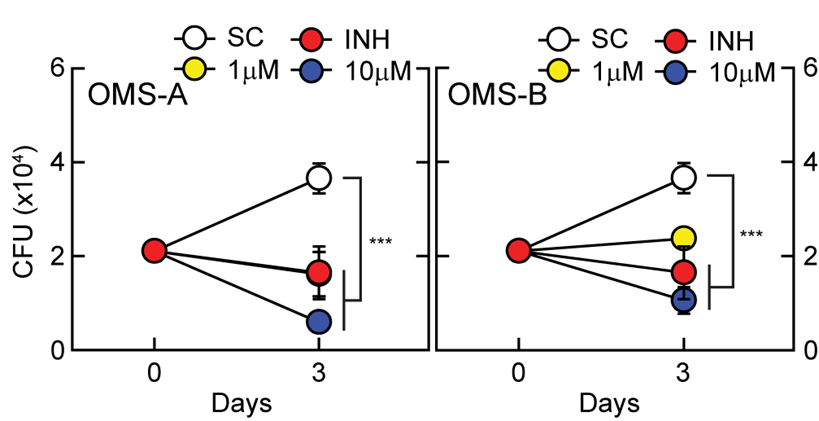
**

**Supplementary Figure S1. OMS reduces Mtb survival in macrophages.** BMDMs were infected with Mtb (moi = 1) for 4 h and then treated with OMS-A (1, 10 μM), OMS-B (1, 10 μM), or INH (0.5 μg/ml). After 3 days, the intracellular bacterial loads were determined using CFU assays. All data represent the means ± SD of triplicates from each sample. ***p < 0.001, compared with SC. SC, solvent control; INH, isoniazid.

**Supplementary Figure S2. OMS-induced autophagy is comparable to those with other autophagy activators.** BMDMs were treated with rapamycin (200 nM for 4h), Torin1 (10 μM for 6 h), OMS-A (10 μM for 24 h), or OMS-B (10 μM for 24 h). (a) Representative confocal microscopic images from three independent samples are shown. Scale bar, 5 μm. (b) Quantitative analysis of LC3 puncta per cell. Image analysis was performed at least 100 cells scored from 6 random fields. Data shown are from one representative of at least three independent experiments (means ± SD of triplicates [b] samples). ***p < 0.001, compared with untreated control (b). U, untreated; Rapa, Rapamycin.

**Supplementary Figure S3. OMS treatment induces autophagy in macrophages**. BMDMs were treated with OMS-A (10 μM; a) or OMS-B (10 μM; b) for the indicated times (0-48 h). LC3 and actin levels were analyzed in cell lysates by immunoblotting. Data shown are from one representative of at least three independent experiments.

**Supplementary Figure S4. OMS-mediated phagosomal maturation depends on the activation of autophagy in macrophages.** (a-d) *Atg7*^fl/fl^ LysM-Cre^-^ and *Atg7*^fl/fl^ LysM-Cre^+^ BMDMs were infected with ERFP-Mtb (moi = 10) for 4 h and then treated with OMS-A (10 μM) or OMS-B (10 μM) for 24 h. (a) Mtb-ERFP (red), Alexa 488-conjugated-LC3 (green), and DAPI (blue) were detected by confocal analysis. Representative confocal microscopic images from three independent samples are shown. Scale bar, 5 µm. (b) Statistical analysis of Mtb-ERFP and LC3 colocalization per cell, with each experiment including at least 100 cells scored from 6 random fields. Data shown are from one representative of at least three independent experiments (means ± SD of triplicates [b] samples). *p < 0.05, ***p < 0.001, compared with control condition. U, uninfected/untreated; SC, solvent control.

**Supplementary Figure S5. LC/MS analysis showing the purities of OMS.** OMS-A (a) and OMS-B (b) were analyzed using a Phenomenex C18 (2) column (100 × 4.6 mm), with a flow rate of 0.7 mL/min, UV detection at 230, 254, 280, and 360 nm, and 10–100% acetonitrile/water with 0.1% formic acid over 20 min.

**
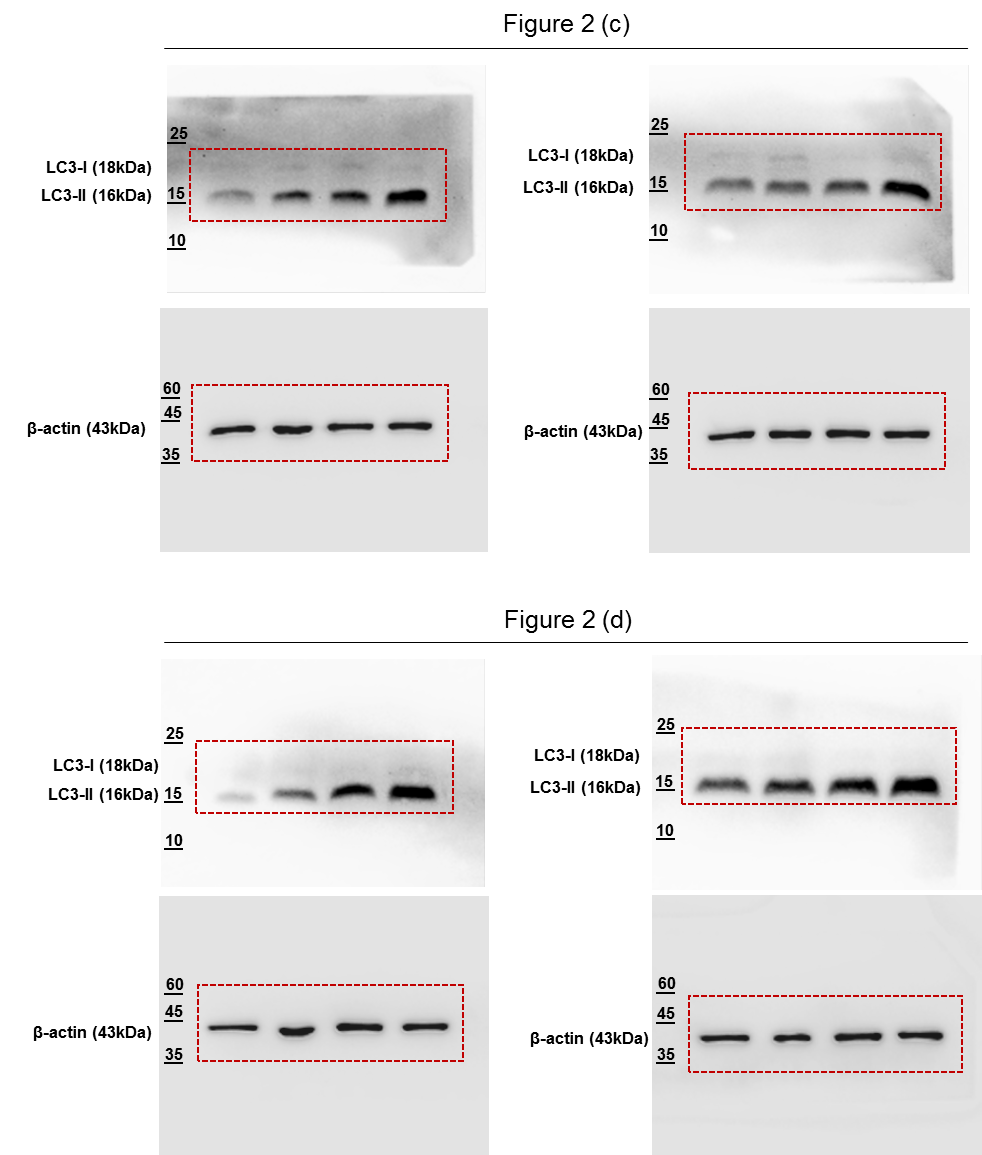
**

**Supplementary Figure S6. Full-length blots in the main paper are presented.**

**Uncropped blots from Figure 2.**

**
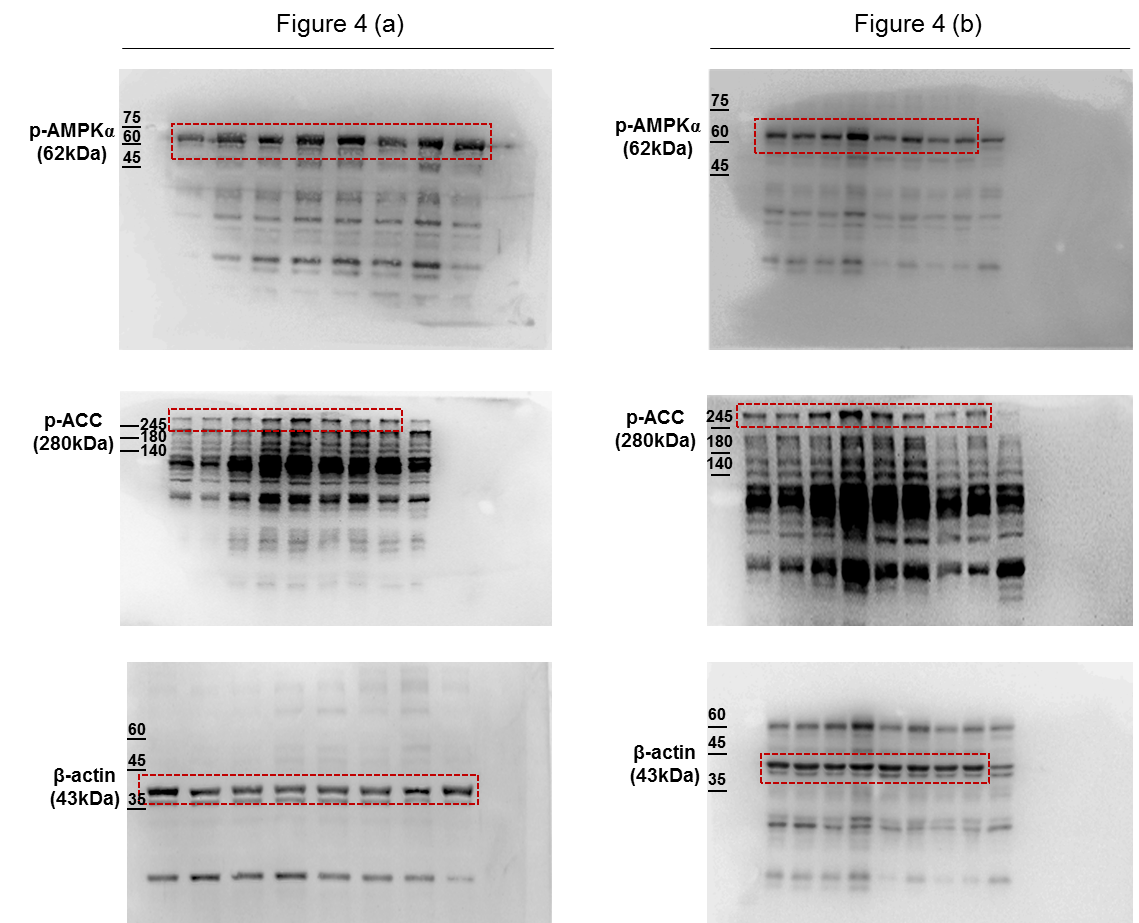
**

**Supplementary Figure S7. Full-length blots in the main paper are presented.**

**Uncropped blots from Figure 4.**
